# Apoptotic functions of microRNAs in pathogenesis, diagnosis, and treatment of endometriosis

**DOI:** 10.1186/s13578-020-0381-0

**Published:** 2020-02-11

**Authors:** Mona Taghavipour, Fatemeh Sadoughi, Hamed Mirzaei, Bahman Yousefi, Bahram Moazzami, Shahla Chaichian, Mohammad Ali Mansournia, Zatollah Asemi

**Affiliations:** 1 Department of Gynecology and Obstetrics, Ramsar Campus, Mazandaran University of Medical Sciences, Sari, Iran; 2 Research Center for Biochemistry and Nutrition in Metabolic Diseases, Kashan University of Medical Sciences, Kashan, Islamic Republic of Iran; 3 Stem Cell Research Center, Tabriz University of Medical Sciences, Tabriz, Iran; 4 Pars Advanced and Minimally Invasive Medical Manners Research Center, Pars Hospital, Iran University of Medical Sciences, Tehran, Iran; 5 Department of Epidemiology and Biostatistics, School of Public Health, Tehran University of Medical Sciences, Tehran, Iran

**Keywords:** Apoptosis, miRNA, Endometriosis, BCL-2, BAX

## Abstract

MicroRNAs or miRNAs are a component of the non-coding RNAs family which is engaged in many cellular functions such as cell proliferation, apoptosis, gene expression, signaling pathways, angiogenesis, and etc. Endometriosis is a malignant gynecologic disorder occurring in women before menopausal age. Pathogenesis of this illness is still a discussion subject between the scientists but in our knowledge, microRNAs can be one of the possible involved factors. The purpose of this paper is to investigate the role of apoptotic activities of miRNAs in endometriosis. Accumulative evidence has demonstrated the role of cell proliferation, apoptosis, and invasion in the progression of these diseases. In this review, we looked into the specific role of apoptosis and its related genes and pathways in endometriosis and tied to present an explanation of how miRNAs can affect endometriosis by their apoptotic activities. What we found is that a great extent of miRNAs is involved in this illness and they are responsible for repressing apoptosis and progression of the disease. As a result, miRNAs have two different usages in endometriosis: biomarkers and potential therapeutic targets. In this review we gathered a great amount of evidence to inquire into the role of micro RNAs in inducing apoptosis and how this mechanism can be exerted for therapeutic purposes for endometriosis.

## Background

Generally, RNAs which are transcribed from the non-coding parts of the genome can be classified into three classes: housekeeping RNAs, transfer RNAs, and regulatory RNAs. MicroRNAs (miRNAs) are a kind of small regulatory RNAs and are approximately consist of 22 nucleotides [1, 2]. RNA polymerase II transcripts these single-stranded RNAs from DNA, then they get processed in the nucleus and after moving to cytoplasm, their maturation starts [3]. MiRNAs after completing the maturation process by Dicer (RNase III) [4], bind to some proteins and produce a ribonucleoprotein complex which is involved in silencing the genes and because of that it’s called RNA-induced silencing complex (RISC) [2]. miRNA binds to the 3′ untranslated region of the target mRNA and takes its part as a down-regulator for gene expression by inhibiting the starting of translation or by deterioration of mRNA [5]. Because of this important function of miRNAs’, they are involved in some crucial processes including: maintenance of stem cells, developmental timing, metabolism, the interaction between a virus and its host, apoptosis, proliferation of the cardiac and skeletal muscular cells and expression of the genes related to neuronal system [6, 7].

Every month through the menstrual period, endometrium gets prepared for the implantation of embryo by the means of bearing a class of biological alterations. Inflammatory reactions, programmed cell death, proliferation of the cells, angiogenesis, tissue formation or differentiation, and remodeling of the tissues are some examples of the engaged processes in altering the endometrium. These processes are regulated by two elements: sex steroids secreted from ovaries and products of the expression of the local genes. There are also some other regulators which their secretion can be autocrine or paracrine and include several growth factors, cytokines, chemokines, proteases, and extracellular matrix [8–11]. Because of the precise expression of this significant regulators, any disturbance can cause an improper regeneration in the endometrium tissue and this may lead to some other abnormalities such as endometriosis [8].

Endometriosis is a condition known by ectopic endometrial glands and stroma which has a dependency to estrogen and is counted as an inflammatory disorder. These ectopic glands and stroma are frequently found in pelvis but there are some other locations in which they have been observed: the bowel, diaphragm, umbilicus, and pleural cavity.

According to evidence, three subtypes are observed for endometriosis: superficial injuries in peritoneal area, deep penetrating wounds, and cysts or endometriomas which are consist of blood and endometrium-like tissue [12]. Dysmenorrhea, pelvic pain, urinary tract symptoms, and rectal bleeding are some symptoms by which endometriosis is diagnosed [13]. Endometriosis, by dint of hormonal induction, stimulation of neural pathways, inflammatory processes, and local bleeding, is able to cause pain but the exact mechanisms are not clear [12]. One of the most important causes of endometriosis is infertility. According to the researches, 25–50% of women with infertility are diagnosed with endometriosis and also 30–50% of women with endometriosis are estimated to be infertile [14]. It is also worth to mention that some studies declared that some of the lesions caused by endometriosis, especially the ovarian ones, appear to be monoclonal and being monoclonal is commonly known as a hallmark for neoplasia [15]. Therefore, endometriosis, because of its unfavorable impacts on quality of life, work productivity, and fertility status, is an important disorder among the women in reproductive age [16]. In this review we gathered a great amount of evidence to inquire into the role of micro RNAs in inducing apoptosis and how this mechanism can be exerted for therapeutic purposes for endometriosis.

## Endometriosis pathogenesis

Generally, we put the name of endometriosis on an inflammatory estrogen-dependent condition in which endometrial-like tissues are found in some places other than its real position. Till now, scientists have not come to an agreement about the mechanism by which this condition is caused [17]. Some researchers have come up with some theories such as retrograde menstruation, coelomic metaplasia, and genetic alterations but still, further investigations are needed for clarifying the exact mechanisms. Observing the volume of blood and fragments of endometrial tissue which is refluxed during a menstrual period in women with endometriosis led to the retrograde menstruation theory. According to this theory, these retreated endometrial fragments can be implanted into some other site than uterine and then grow into an ectopic endometrial-type tissue [18–20]. In another theory, the reason of this ectopic tissue’s production is mainly attributed to metaplasia of the coelomic epithelium which itself is caused by environmental factors. As well, some scientists believe that the anatomical position of uterine, lymphatic or hematogenous spread of endometrial-related cells, and some other factors such as proton irradiation and dioxin might play a role in pathogenesis of endometriosis [21].

In the immunity point of view, there is also a theory regarding the role of deficient immunity in endometriosis. Haptoglobin and monocyte chemoattractant protein 1 are two agents that might take part in causing endometriosis. In addition, some evidence accounts the abnormal B cell, T cells, or natural killer cells as the responsible factor for this gynecological disease. Moreover, inflammation caused by increased amounts of prostaglandins and cytokines is another possible reason that causes endometriosis [17, 18].

In addition, Arvanitis et al. [22] showed the relationship between CYP1A1, CYP19, GSTM1, and GSTT1 polymorphisms and endometriosis.

## Apoptosis: a caspase-dependent process

Generally, programmed cell death or PCD was first separated from apoptosis when necrosis was discovered as another pattern for cell death in early 1970s [23]. Apoptosis is an active exclusive molecular process which is essential in animals with a great number of cells and helps them to regulate the size of their tissues by modulating the number of their cells and also keeps them protected from the cells which can be threats to their homeostasis [24]. Any alteration in the mechanism of apoptosis can cause a complication: Hyper activation of apoptosis leads to some conditions like neurodegenerative diseases, deficiency of the immune system, and ischemia–reperfusion injury and also the suppression of apoptosis can result in some other conditions including cancer, and autoimmune diseases [25].

A group of considerable morphological and biochemical alterations happen through this process and thereby cells first shrink and their nuclei condense, and then they disintegrate into well-enclosed apoptotic bodies [26]. In a simple model, three phases can be named for apoptosis: initiation by two different mechanisms, genetic regulation by pro and anti-apoptotic genes, and effector mechanisms by caspases [27]. Commonly, there are two mechanisms that initiate apoptosis: extrinsic and intrinsic pathway. These two pathways are dependent to a group of homogeneous cysteine proteases named caspase family. Caspases are cell-killer proteases and till now, 10 different kinds of them are discovered [28–30].

In the intrinsic pathway, two groups of mitochondrial proteins enter the cytosol in response to stimuli such as hypoxia, free radicals, and radiation. The first group of these proteins causes activation in the caspase-dependent mitochondrial pathway which leads to the activation of caspase-9. The other group causes a fragmentation and a condensation in the DNA and peripheral chromatin of the nuclei, respectively [29, 31–33]. The extrinsic pathway, despite the intrinsic one, what stimulates the process is a ligand and therefore this pathway is dependent to some receptors named death receptors. After the attachment of some ligand such as FasL, TNF, and TRAIL to the death receptors, a death signal is sent to the intracellular pathways and causes the formation and activation of a death-inducing signaling complex or DISC and caspase-8, respectively [33–36]. In the second phase, a motif of gene expression is created through a diversity of mechanisms by the initiators. There are two kinds of these expressed genes: pro-apoptotic (death genes) and anti-apoptotic (survival genes). Two major protein families expressed by these genes are BCL-2 and p53 families. The BCL-2 is a family of proteins which has more than 20 members and includes both survival and death proteins [37, 38]. The most known genes of this family are BCL-2 itself, Bim, and the gene BAX which are respectively, anti-apoptotic, pro-apoptotic, and pro-apoptotic [39, 40].

## MicroRNAs and apoptosis

As mentioned before, miRNAs are small evolutionary conserved RNAs which are able to alter the gene expression by affecting the gene’s related mRNA [5]. It is estimated that miRNAs are responsible for more than 60% of gene regulations (coding genes). As well, these RNAs are able to suppress or induce apoptosis by altering its regulatory genes and thus take part in both intrinsic and extrinsic pathways. MiRNAs can influencing the gene expression in two ways: affecting mRNAs directly or indirectly by affecting other miRNAs [41, 42]. In extrinsic pathway, some miRNAs are reported to alter the expression of related ligands. For example, miR-21 is able to directly inhibit FasL and increase apoptosis or miR-130a is able to decrease the TRAIL resistance by the means of affecting some other miRNAs and augment apoptosis [43, 44].

Because of the important role of p53 and BCL-2 families in regulating the intrinsic pathway, miRNAs can impact intrinsic pathway by changing the expression of the proteins of this two families [42]. Accumulating evidence demonstrates that more than 20 kinds of miRNAs are directly regulating the pro-apoptotic p53 gene by binding to the 3′ untranslated region of its mRNA. For instance, one of these miRNAs which can be produced by cancer cells is miR-504. These miRNA binds to two sites of the 3′-UTR region of p53 mRNA and therefore, causes the down-regulation of this gene [38, 45, 46]. Additionally, miRNAs can also take part in changing the expression of apoptotic caspases such as caspase-9 and caspase-3 which are blocked by miR-23a and miR-421, respectively [47, 48]. Therefore, miRNAs are involved in almost all the phases of apoptosis and are able to reduce or induce a cell’s apoptosis by influencing intrinsic pathway, extrinsic pathway, and cascades. On the other hand, miRNAs can be used for diagnosis and treatment of many diseases such as cancer and even for prevention of some diseases (according to their potential for pathogenesis).

## Apoptosis and endometrium: normal status

We can name three stages for the cycle of endometrium in menstruating women: proliferating, secretory, and menstrual stages [49]. The presence of apoptosis has been detected (in great extents) in the late secretory, menstrual, and (in very low extents) proliferative and early secretory stages [50–52]. Considering the role of estrogen and progestin in different phases of this cycle, creates this idea that they also have a part in regulating the apoptosis process. Vaskivuo et al. [53] showed that in the proliferative stage, there is an association between the concentration of estradiol in serum and down-regulation of apoptosis. Another involved factor in regulating the apoptosis during the phases of endometrial cycle is the BCL-2 protein produced from its gene. In endometrium, glandular and stromal cells express BCL-2 [54]. Otsuki et al. [55] revealed that during the proliferating time, BCL-2 holds back the apoptosis and hence, it’s increased at the end of the first phase and reduced through the late secretory and menstrual stages. In spite of the expression amounts of BCL-2 in glandular cells, consistent immunoreactivity of this gene was observed in smooth muscles of the myometrium layer of the uterine [56]. This evidence demonstrates that the product of BCL-2 gene not only protects the glandular cells of endometrium but it also is important in the survival of smooth muscles of myometrium. In addition, Rogers et al. found that basal layer of the endometrium, in spite of the functional layer, shows more expression of BCL-2 gene and it’s because this layer should be constant through the endometrial cycle. They also revealed that steroid hormones are one of the effective factors on altering the BCL-2 expression [57].

There also some other members of the BCL-2 protein family that studies has showed their impact on up or down-regulating the apoptosis in the different layers of endometrium during the endometrial cycle: BAX [51], BCL‐X [58], and BAK [59]. These mentioned proteins do their duty in mechanisms which are dependent or independent from the action of BCL-2 [60]. All this together, these data insinuate that there is a dynamic cooperation among a considerable number of members of the BCL-2 family which leads to initiation of apoptosis [60] and ovarian steroids may have a part in apoptosis by controlling the expression of this members of BCL-2 family [55]. Another piece of machinery that is engaged in managing the apoptosis is Fas/FasL system. Fas is a member of TNF/nerve growth factor receptor family which has 45 kDa weight and is counted as a type I protein of the cell membrane [61]. FasL is the ligand of the Fas protein and is a 37 kDa protein. The interaction of Fas and its ligand is a key to the induction of apoptosis [62]. In the course of late proliferative stage, these proteins are being conserved in the Golgi apparatus and cytoplasmic vesicles but they are not able to interact; while in the second phase their release turns on the apoptosis [63]. Membrane‐bound and soluble are two kinds of FasLs. Some matrix metalloproteinases or MMP turn the first type into second type which is the active form [64]. In the endometrium, augmented function of MMPs before and throughout the menstruating stage (type 1, 3, and 9) [65] and during the secretory phase [66]. Moreover, BCL-2 also has a role in managing the Fas/FasL related apoptosis: BCL-2 inactivates an enzyme called interleukin-converting enzyme or ICE and thereby inhibits this pathway of apoptosis [67].

## Apoptosis and endometrium: abnormal status of endometriosis

**Fig. 1:**
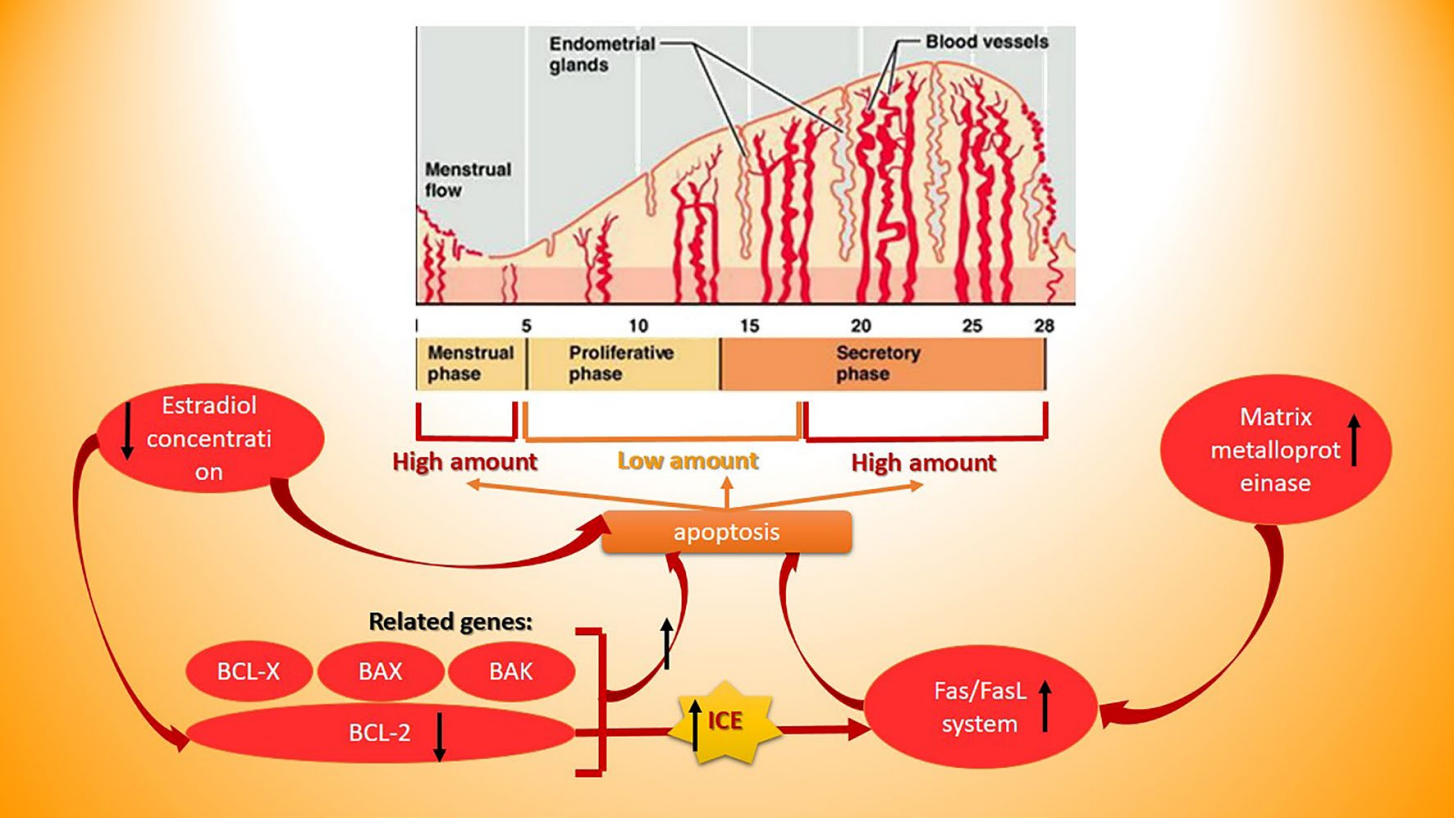
Endometriosis and related factors and miRNAs

In endometriosis, not only the ectopic endometrium-like tissue goes through a bunch of alterations but the eutopic endometrium also have some differences with the normal one [60]. These differences are consist of a diversity of abnormalities in structure, cell proliferation, immune elements, proteolytic enzymes and their inhibitors, production of steroids and cytokines and proteins, and expression of different genes [68]. These differences can be a factor in the survival of the regurgitating endometrial cells into the peritoneal cavity and thus help the progression of endometriosis [60]. Recently, the presence of apoptosis detected in women with endometriosis has been significantly considered. The number of entered survival cells into the peritoneal cavity is increased in these women because of the reduced amounts of apoptosis during the late secretory/menstrual and early proliferative stages [69]. Expression of the proteins of the BCL-2 family and Fas proteins are the probable factors responsible for diminution of apoptosis in this disorder. Watanabe et al. [54] compared the eutopic and ectopic endometrial tissues in women with endometriosis and found that in spite of the ectopic tissue, expression of BCL-2 has a cyclic pattern in glandular cells of the eutopic tissue. Jones et al. [70] also observed an overexpression of BCL-2 in stroma cells of the ectopic tissues (which might be related to the augmented number of estrogen receptors of the stromal cells [71]). Another research conducted by Meresman et al. [72] manifested that in the first phase of apoptosis expression of BCL-2 and BAX are respectively elevated and absent and BAX is also overexpressed in the second phase in women with this disease compared to the healthy ones (Fig. 1).

We did not found any study comparing the expression of Fas in women with and without disease but a plenty of studies showed that the expression of FasL is higher in women having the disease [73]. Furthermore, some growth factors released from macrophages like platelet-derived growth factor (PDGF) and transforming growth factor (TGF) are observed in peritoneal fluid of ill women more than disease-free women. These two growth factors stimulate stromal cells to express more FasL, thus, peritoneal macrophages also contribute to increase the apoptosis of immune cells. It means that ectopic endometroic cells express more FasL to protect themselves against the T cells of immune system [74, 75]. Additionally, higher extents of MMPs have been seen in sick women who can have a hand in decreasing the immune attacks by T lymphocytes of the host [76–78]. Another observed increased agent in the women with endometriosis is IL-8 which is a chemokine inducing the stromal cell’s proliferation and thereby aids the endometroic tissue to grow. Selam et al. [79, 80] found that IL-8, as well, is instrumental in elevating the immunotolerance by the means of stimulating production of FasL. In addition to all of that, even the expression of BCL-2 and BAX is changed in peritoneal macrophages during the endometriosis and so the process of apoptosis is delayed and resistance of disease to immune system is increased [70, 81]. All this considered, apoptosis is a key player in pathophysiology of endometriosis.

## MicroRNA and endometriosis: apoptotic activities

As mentioned before, miRNAs are able to affect apoptosis in many ways: they can regulate each step of apoptosis in both stimulatory and inhibitory manners. As a result, not only miRNAs are involved in the pathogenesis of several diseases but they also have the potential of being used as biomarkers or therapeutic targets. Considering the pivotal role of apoptosis in endometriosis, we suggest that these RNAs might start a revolution in our understanding of endometriosis pathogenesis and help us provide more efficient diagnostic and therapeutic procedures. Several kinds of research are conducted about different miRNAs involved in endometriosis by regulating apoptosis which we are going to represent some of them in this section.

Zhang et al. [82] investigated the role of miR-141-3p in endometriosis and demonstrate that this miRNA is down-regulated in ectopic endometrium. MiR-141-3p is one of many miRNAs which affects apoptosis by reducing and inducing the expression of BCL-2 and Bax, respectively. This means that not only this miRNA can be considered as a pathogenesis-related factor but it also can be used as a biomarker and a therapeutic target for endometriosis. MiR-9 is another miRNA that is able to target the BCL-2 mRNA and thereby induce apoptosis. Dysregulation of this miRNA has been observed in some other gynecological diseases like ovarian cancer, as well. According to the down-regulation of this miRNA in endometriosis, Burney and colleagues suggest that this miRNA is somehow related to endometriosis pathogenesis [83]. Similarly, there are some other miRNAs (summarized in Table 1) which their association to endometriosis has been examined and therefore, they have given us a new insight for the pathogenesis of this diseases. Furthermore, they are capable of taking part in treatment of endometriosis by targeting the oncogene mRNAs or by being the target of anti-cancer drugs.

**Table 1:** Experimental studies that investigated the role of miRNAs involved in regulating apoptosis in endometriosis

microRNA	Expression	Function	Application	References
miR-33b	Down-regulated	Facilitating caspase 3 activity	Treatment and biomarker	[96]
miR-191	Up-regulated	Inhibition of TNF-alpha induced apoptosis and might contribute malignant transformation of endometriosis	Treatment and biomarker	[97]
miR-503	Down-regulated	Targeting BCL-2 mRNAs	Treatment and biomarker	[98]
miR-183	Down-regulated	Reducing apoptosis	Treatment and biomarker	[99]
miR-29c	Down-regulated	Targeting c-JUN (a protein which regulates gene expression)	Treatment and biomarker	[100]
miR-148a	–	Affecting BCL-2 expression and caspase-3/9 through G protein coupled estrogen receptor/miR148a/human leukocyte antigen G signaling pathway	Treatment	[101]
miR-210	Up-regulated	Inhibition of apoptosis by targeting signal transducer and activator of transcription 3	Treatment and biomarker	[102]
miR-196b	–	Affecting BCL-2 expression, activation of caspase-3 and caspase-7	Treatment	[103]
miR-363	Down-regulated	Inducing apoptosis	Treatment and biomarker	[104]
miR-21-5p	–	Affecting the apoptotic potential of stromal cells.	Treatment	[92]
miR-9	Down-regulated	Affecting BCL-2 expression	Treatment and biomarker	[83]
miR181c	–	NR4A-miR181c-Mst1 pathway regulates mitochondrial apoptosis	Treatment	[105]
miR-2861	Down-regulated	Up-regulation of STAT3 and MMP2 and inducing apoptosis	Treatment and biomarker	[106]
miR-141-3p	–	Down-regulation the expression of Bcl-2 and raising the expression of Bax and contributing to the apoptosis of ectopic ESCs	Treatment	[82]

## MicoRNA and endometriosis: where are we standing?

Considering the differences existing between the expressed miRNAs of eutopic and ectopic endometrial tissue, miRNAs have the potential to be used as biomarkers in endometriosis [84]. A study showed that in endometriosis cases, miR-34c-5p, miR-9, miR-34b are down regulated in comparison to normal cases [83]. In addition, miR-483-5p and miR-629-3p are also two other miRNAs which their dysregulation in endometriosis has been confirmed [85]. Moreover, there are also several other miRNAs which are related to angiogenesis, inflammatory, cell proliferation, Steroidogenesis, and other mechanisms engaged in endometriosis and are being used as efficient biomarkers for the endometriosis diagnosis [84, 86–88]. However, involved miRNAs in apoptosis are not considered well in the field of diagnosis and thus, we suggest that investigating the efficacy of some miRNAs such as miR-21, miR-155, and miR-a125b is needed.

In treatment point of view, there are some studies that used miRNAs for targeting different genes and signaling pathways engaged in apoptosis. for instance, adammek et al. [89] tried on miR-145 on eutopic and ectopic endometrial stroma cells and found it useful. Another trial used miR-10b to target syndecan-1 to reduce the cell invasiveness and observed considerable results [90]. As well, some other studies used miRNA such as miR-135a/b as therapeutic targets to treat the infertility caused by endometriosis [91]. There is also another trial that examined saponin, a component of Korean red ginseng, and found that it can target miR-21-5p and thereby be a therapeutic tool for endometriosis [92].

Notwithstanding all these evidence, there are still some obstacles in the way of using miRNAs as a common treatment for endometriosis. Low stability and potential side effects are two major limitations of using these RNAs. Furthermore, the lack of proper route of administration and delivering approaches are some other barriers which nanotechnology is removing them out of the way by providing novel delivery systems [93]. All taken together, there is a lot of room in the field of using miRNAs for diagnosis and treatment of endometriosis for further investigations.

## Conclusions

For years, scientists thought that 98% of our DNA was junk and not useful at all [94] but discoveries about the functions of RNAs transcribed from the non-coding parts of DNA made a revolution in our therapeutic and diagnostic approaches for many diseases. miRNAs are a kind of small non-coding RNAs which are known to have many parts in cellular processes (including apoptosis, cell proliferation, and inflammation) by regulating the gene expression. Hence, their contribution in pathogenesis of many diseases such as endometriosis is proven. Endometriosis is a gynecological disorder among the women in reproductive age that might lead to cancer (in a very low percentage of women) and is also an important disease for causing several health problems such as infertility. Accumulative evidence has demonstrated the role of cell proliferation, apoptosis, and invasion in the progression of these diseases. In this review, we looked into the specific role of apoptosis and its related genes and pathways in endometriosis and tied to present an explanation of how miRNAs can affect endometriosis by their apoptotic activities. What we found is that many miRNAs are involved and they are responsible for repressing apoptosis and progression of the disease. As a result, these miRNAs have the potential to be used in diagnostic and therapeutic decisions adopted for endometriosis. In the field of diagnosis, the current gold standard method for endometriosis is using surgery (laparoscopy) and direct observation [95]. Hence, miRNAs have the potential to be an ideal replacement for this dangerous and high-risk approach and plus, they can act more efficient for early detection of endometriosis. Although considering the many roles and functions that miRNAs have in cellular pathways, more investigations are needed for revealing their side effects after administration. Furthermore, finding proper delivery systems which are able to increase the stability of miRNAs, protect them against degradation inside the body, and deliver them to the specific site of the disease are needed.

## Data Availability

Not applicable.

## Abbreviations

miRNA: microRNA
RISC: RNA-induced silencing complex
PDGF: Platelet‐derived growth factor
TGF: Transforming growth factor
PCD: Programmed cell death
TNF: Tumor necrosis factor
MMP: Matrix metalloproteinase
ICE: Interleukin converting enzyme
DAPK1: Death-associated protein kinase 1
